# Polatuzumab vedotin-R-CHP used for high-risk EBV-negative DLBCL-type post-transplant lymphoproliferative disorder in a long-term kidney transplant recipient: a case report

**DOI:** 10.3389/fonc.2025.1673495

**Published:** 2026-01-08

**Authors:** Xiaoyu Jiang, Chunping Wu, Yuxun Oswald Zhang, Mingqing Luo, Yiwen Qiu, Miao Li, Lianshan Zhan, Daping Zhong

**Affiliations:** 1 Department of Hematology, Guiqian International General Hospital (GIGH), Guiyang, Guizhou, China; 2 Department of Nuclear Medicine, GIGH, Guiyang, Guizhou, China

**Keywords:** diffuse large B-cell lymphoma, immunosuppression, polatuzumab vedotin, post-transplant lymphoproliferative disorder, prognostic factors, targeted therapy

## Abstract

**Background:**

Post-transplant lymphoproliferative disorder (PTLD) is a severe life-threatening complication following solid organ transplantation. Among its subtypes, monomorphic PTLD (mPTLD) is the predominant form, most often presenting as diffuse large B-cell lymphoma (DLBCL). Most PTLD is *Epstein–Barr virus* (EBV) related, and EBV-negative PTLD typically arises later in the post-transplant course.

**Case description:**

We report the case of a 57-year-old female who had undergone kidney transplantation 23 years earlier. Routine examination showed elevated serum creatinine and lactate dehydrogenase (LDH) levels, along with tacrolimus concentrations above the therapeutic range. Although asymptomatic, the patient underwent comprehensive evaluation because of the risk of PTLD and graft rejection. Ultrasonography demonstrated multiple masses in the transplanted kidney, and renal biopsy confirmed EBV-negative diffuse large B-cell lymphoma (DLBCL) with a germinal center B-cell (GCB) immunophenotype. An initial clinical response, including partial tumor regression, was observed following a reduction in immunosuppressive therapy and administration of two doses of rituximab. However, the disease progression was confirmed two months later. The patient subsequently underwent six cycles of Pola-R-CHP (polatzumab vedotin, rituximab, cyclophosphamide, adriamycin and dexamethasone; 21-day per cycle). Positron emission tomography/computed tomography (PET/CT) demonstrated regression of the tumor in the transplanted kidney, with a marked reduction in the maximum standardized uptake value (SUVmax). Despite this favorable response, new lesions were detected in the spinal canal of thoracolumbar spine.

**Conclusion:**

In this case, Pola–R–CHP achieved a favorable initial response, suggesting a potential role in the management of mPTLD. However, the subsequent central nervous system relapse underscores the need for further studies that incorporating CNS prophylaxis to define its optimal application.

## Introduction

Post-transplant lymphoproliferative disorder (PTLD) is a rare but serious malignancy that develops in the context of long-term immunosuppression after solid organ transplantation. Reduction of immunosuppression (RIS) is the first line treatment, followed by rituximab monotherapy for RIS-refractory cases. For patients who fail to respond to RIS and rituximab, or who present with aggressive histological subtypes, chemotherapy such as R-CHOP (rituximab, cyclophosphamide, doxorubicin, vincristine, and dexamethasone) is typically used (1, 2). Polatuzumab vedotin (Pola), an antibody–drug conjugate targeting CD79b, has shown efficacy in DLBCL in patients without underlying immune deficiency. Compared with R-CHOP, the Pola–R–CHP regimen (polatuzumab vedotin, rituximab, cyclophosphamide, doxorubicin, and dexamethasone) significantly improved progression-free survival (76.7% vs 70.2%), leading to its approval as first-line therapy for DLBCL (3, 4). Reports have also described its application in PTLD. In one case, a patient with EBV-positive, double expressor–negative (EBV^+^/DEL^-^) DLBCL (non-GCB, stage IV, IPI 2) achieved complete remission after receiving four doses of rituximab followed by six cycles of the POLA–R–CHP regimen. A liver biopsy performed for imaging abnormalities revealed no definitive evidence of PTLD, and EBV-PCR was undetectable at that time (5). Another report (6) described favorable therapeutic response of DLBCL–type methotrexate-associated lymphoproliferative disorder (MTX-LPD, stage IV, high-risk, non-GCB subtype) with six cycles of the Pola–R–CHP. Although the pathogenesis of MTX-LPD and mPTLD differs, both share a common mechanism of abnormal B-cell proliferation in the setting of immunosuppression. By contrast, Pola-based therapy has shown poor outcomes in patients with high-grade B-cell lymphoma (HGBL-NOS) or *MYC/BCL2*-rearranged tumors (7).

## Case presentation

A 57-year-old female, who had undergone kidney transplantation at the age of 34 for end-stage renal disease secondary to uremia, was maintained on a combined immunosuppressive regimen of mycophenolic acid (500mg twice daily) and tacrolimus (1mg in the morning and 0.5mg in the evening; trough concentration of 5.0 ng/mL). At the time of recruitment, 23 years post-transplantation, routine evaluation revealed elevated serum creatinine (92.3umol/L), lactate dehydrogenase (LDH) levels (509.44U/L), and tacrolimus levels (6.9 ng/mL). EBV-DNA and BKV-DNA PCR tests were negative (40 cycles). Ultrasonography showed multiple masses in the transplanted kidney. Subsequent PET/CT revealed multiple hypermetabolic masses, with the largest measuring 42×26 mm (**Figures 2A, B**). Ultrasound-guided kidney biopsy confirmed EBV-negative DLBCL with GCB immunophenotype.

**Figure 1:**
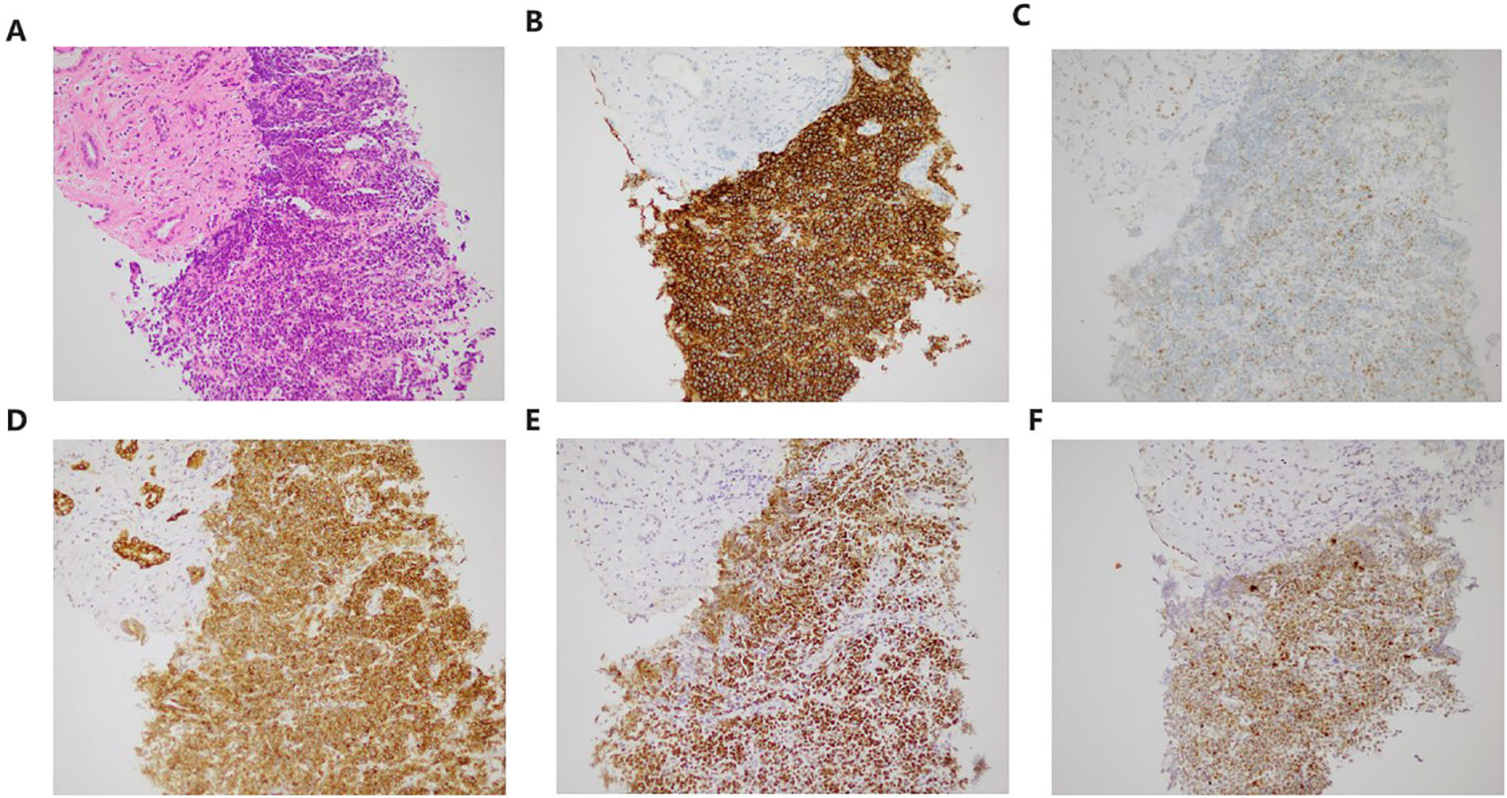
Imaging features of the PTLD. **(A)** H&E staining indicated diffuse proliferation of atypical lymphocytes. **(B-F)** IHC showing tumors positive for CD20, c-Myc (50%), BCL-2 (80%), BCL-6, p53 mutant (80%). All×200 magnifications.

The Ki-67 (**Figure 1E**) index was 90%, indicating high proliferative activity. Immunohistochemical staining was positive (**Figures 1B–F**) for CD20, c-Myc (50%), BCL-2 (80%), BCL-6, and p53 mutant (80%), and negative for CD30 and EBER. These findings confirmed the diagnosis of double expressor/EBV-negative DLBCL. In the context of the patient’s history of renal transplantation and long-term immunosuppression, the diagnosis was established as PTLD. The DLBCL International Prognostic Index (DLBCL-IPI) score was 3, consistent with Stage IV disease due to renal involvement and elevated LDH.

RIS was initiated: mycophenolic acid was replaced with sirolimus (1mg once daily in the morning), tacrolimus was tapered to 0.5mg (morning), and prednisone was maintained at 20 mg/day. Due to an elevated HCV-RNA level (9.96×10^6^ IU/mL), antiviral therapy with sofosbuvir and velpatasvir was administered. Tumor shrinkage was observed following immunosuppression reduction alone.

**Figure 2:**
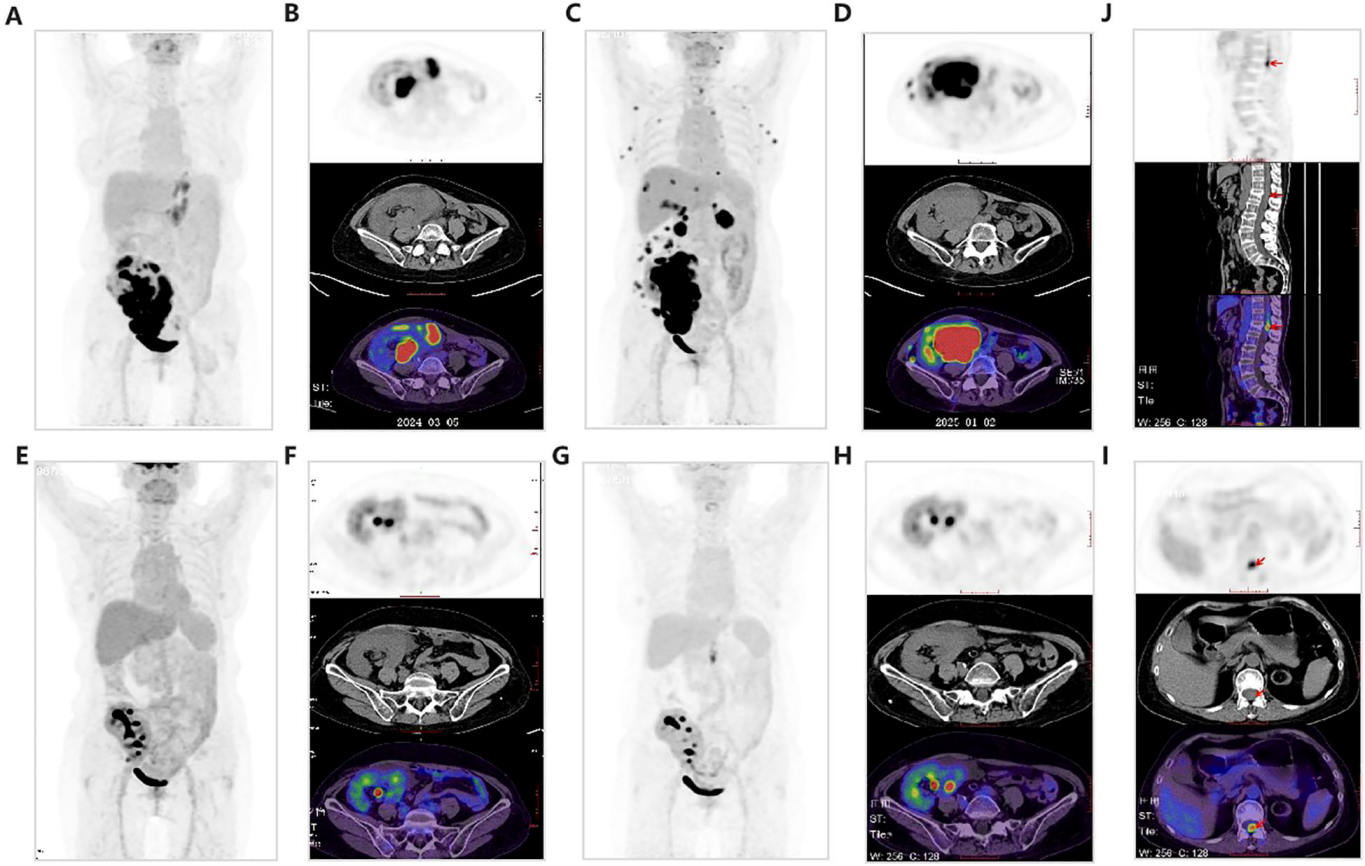
Nuclear medicine imaging. **(A, B)** PET/CT at initial diagnosis. **(C, D)** PET/CT at disease progression, 10 months after diagnosis. **(E, F)** PET/CT after three cycles of Pola-R-CHP, 13 months after diagnosis. **(G–J)** PET/CT at 17 months after diagnosis, two months after completion of six cycles of chemotherapy.

At two and eight months after diagnosis, two doses of rituximab were administered alongside continued RIS. Because the patient’s B lymphocyte count was already markedly reduced, the nephrologists decided not to complete the standard 4–6 dose course of rituximab to reduce the risk of severe infection. At ten months after diagnosis, the patient developed gross hematuria, a marked increase in serum creatinine (418.2 μmol/L), and progression of the transplanted kidney tumor. DNA quantification for *hepatitis C Virus* (HCV), *cytomegalovirus* (CMV), and EBV was negative. A repeat PET/CT at the same time confirmed tumor progression, with the largest lesion measuring 68 × 74 mm and a maximum standardized uptake value (SUVmax) of 69.7 (**Figures 2C, D**). Supplementary fluorescence *in situ* hybridization (FISH) showed no rearrangements in *BCL-2*, *BCL-6*, or *c-MYC* but confirmed deletion of the *TP53* gene. No lymphoma cells were found in bone marrow cytology or histopathology. The diagnosis was stage IV DLBCL (IPI score 3, GCB subtype, double expressor). Additional findings of *TP53* deletion indicated high-risk disease features.

Tacrolimus and sirolimus were discontinued, and methylprednisolone (40 mg/day) was initiated. After five days, serum creatinine decreased to 127 μmol/L and hematuria resolved. The patient was classified as high risk according to the Central Nervous System International Prognostic Index (CNS-IPI ≥4), but given the severity of the PTLD and the potential risks of prophylactic therapy, intrathecal methotrexate was not administered. At eleventh months after the diagnosis, the patient began Pola-R-CHP treatment. Immunosuppressants were withheld during chemotherapy, but tacrolimus, azathioprine and prednisone were reintroduced following treatment. After three cycles of Pola-R-CHP, serum creatinine decreased to 96.24 μmol/L, and PET/CT demonstrated a reduction in SUVmax from 69.7 to 7.3 with a Deauville 4-point scale (**Figures 2E, F**). The next three cycles of Pola-R-CHP were successfully completed. However, due to weakness, the patient was not followed up regularly and could not receive maintenance therapy. At 17 months after diagnosis (2 months after completion of six cycles of chemotherapy), the patient developed sudden lumbosacral pain radiating to both lower limbs, which worsened at night and required oral analgesics. Physical examination revealed localized lumbar tenderness, restricted lumbar motion, a positive straight leg raise test bilaterally, preserved muscle strength (grade 5), and no swelling. PET/CT showed that the largest lesion had decreased to 15 × 16 mm with an SUVmax of 5.2 (**Figures 2G, H**), but new hypermetabolic lesions were simultaneously detected in the spinal canal, with an SUVmax of 11.3 and a Deauville score of 5 (**Figures 2I, J**).

## Discussion

Initial management of PTLD typically involves gradual RIS, as early lesions may resolve with this approach (8). In cases refractory to RIS, rituximab—an anti-CD20 monoclonal antibody widely used for CD20-positive lymphomas—can be administered as monotherapy or in combination with chemotherapy. Single-agent rituximab is often preferred initially, with chemotherapy reserved for patients who fail to respond. Approximately 20% of patients with PTLD achieve a complete response with rituximab monotherapy (9), and its adverse effects are generally more tolerable than those associated with combination chemotherapy. On the other hand, combining rituximab with chemotherapy has been shown to lower the risk of organ impairment, particularly in renal transplants recipients (10). There are high quality data and clinical trials supporting the use of R-CHOP in patients with rituximab failure (1, 2). When rituximab is combined with chemotherapy, complete response rates increase to 65% (2). Notably, in some refractory cases, Pola has been shown to enhance CD20 expression and increase sensitivity to rituximab-induced complement-dependent cytotoxicity (CDC) in several Pola-refractory cells. In xenografted models, Pola similarly increased CD20 expression and significantly augmented antitumor activity when combined with rituximab (11).

Since Pola has significantly improved progression-free survival (PFS) in previously untreated DLBCL, as demonstrated in the phase III POLARIX study (12), it may represent a promising therapeutic option for refractory PTLD. The patient demonstrated good tolerance to Pola-based regimen, and the treatment course proceeded smoothly. The observed clinical efficacy suggests that Pola-R-CHP may enhance therapeutic outcomes for PTLD.

However, in specific populations such as mPTLD, high-risk features—including double expression, p53 protein mutation, and *TP53* gene deletion—warrant close attention. Although *TP53* mutations are among the most common genetic alterations in human tumors, their prevalence in lymphomas is relatively low (approximately 20%). In this patient, 80% of tumor cells exhibited strong p53 protein expression, strongly suggesting a *TP53* missense mutation, which is typically associated with greater invasiveness, treatment resistance, and poorer outcomes. The prognostic significance of *TP53* gene deletion detected by FISH remains controversial. While some studies have reported that *TP53* gene deletion is an adverse prognostic factor in DLBCL (13, 14), others have found no significant association with overall survival (15). Double expression of MYC and BCL-2 in DLBCL is also recognized as an adverse prognostic marker. When double-expressor DLBCL occurs with a high Ki-67 index and non-GCB subtype, the clinical course is particularly unfavorable and often necessitates more intensive therapy (16). In our patient, intraspinal relapse occurred 17 months after diagnosis (two months after completion of six cycles of chemotherapy). These observations suggest that, in cases with high-risk features such as double expression and *TP53* mutation, strategies to prevent central nervous system involvement should be considered, even when Pola–R–CHP is used as the treatment regimen. Accumulation of additional cases and careful evaluation of individual disease courses will help inform diagnostic decisions and optimize outcomes in similar patients.

## Conclusions

This case suggests that Pola-R-CHP can induce a favorable initial response in PTLD with multiple high-risk features, indicating its potential as an alternative option in challenging cases. However, the subsequent development of intraspinal lesions calls for careful consideration of central nervous system prophylaxis. These findings indicate that further investigation is warranted to clarify the role of Pola-R-CHP in the management of PTLD and to define its long-term efficacy and optimal use.

## Data Availability

The original contributions presented in the study are included in the article/supplementary material. Further inquiries can be directed to the corresponding author.
